# Evaluation of the Healthy Schools Program: Part I. Interim Progress

**DOI:** 10.5888/pcd9.110106

**Published:** 2012-03-01

**Authors:** Margaret Beam, Ginny Ehrlich, Jessica Donze Black, Audrey Block, Laura C. Leviton

**Affiliations:** RMC Research Corporation, Portland, Oregon; Alliance for a Healthier Generation, Portland, Oregon; Pew Charitable Trusts, Washington, DC; RMC Research Corporation, Portland, Oregon; The Robert Wood Johnson Foundation

## Abstract

**Introduction:**

Federal and state policies identify schools as a setting to prevent childhood obesity, but schools need better health-promoting strategies. The objective of this study was to evaluate interim progress in schools receiving hands-on training from the Healthy Schools Program, the nation's largest school-based program aimed at preventing childhood obesity. The 4-year program targets schools with predominantly low-income, African American, or Hispanic students.

**Methods:**

In 2010 we assessed schools that enrolled in the 2007-2008 and 2008-2009 school years. School representatives completed an inventory of 8 content areas: policy and systems, school meals, competitive foods and beverages, health education, physical education, physical activity outside of physical education, before- and after-school programs, and school employee wellness. schools' baseline inventory was compared by *t* test with the most recent inventory available.

**Results:**

Schools made significant changes in all content areas, and effect sizes were moderate to large.

**Conclusion:**

Participating schools improved environmental policies and practices to prevent childhood obesity. The program is a resource to implement recent federal and state policies.

## Introduction

Childhood overweight and obesity are serious problems for the United States, where prevalence has nearly doubled during the past 20 years (1). More than 95% of school-aged youth in the United States attend school, and it is the place where children and youth spend the most time outside the home (2). As a result, national, state, and local policy makers focus on the schools for obesity prevention. In 2010, the report to the President from the White House Task Force on Childhood Obesity (3) and the passage of the Healthy, Hunger-Free Kids Act (4) continued the policy momentum created with passage of the Child Nutrition and WIC Reauthorization Act of 2004 (5). The 2004 act required that all school districts participating in the federally funded school meals programs develop a school wellness policy addressing school nutrition and physical activity (5).

Although the policy momentum for school wellness efforts is clear, research suggests that the 2004 law had modest effects at best (6). Local district policies varied greatly in comprehensiveness, and several studies found no significant changes in schools' nutrition and physical activity environments after passage of these policies (7-10). Given that the Healthy, Hunger-Free Kids Act requires most public school districts to continue the wellness efforts mandated by the 2004 law, we sought to identify strategies that will help promote these efforts in schools.

The objective of this study was to evaluate the progress of schools participating in the Healthy Schools Program (HSP) as they implement health-promoting policy, practice, and environmental changes. The 4-year program targets schools with predominantly low-income, African American, or Hispanic students. HSP is the largest program in the nation devoted specifically to school-based obesity prevention. HSP is part of the Alliance for a Healthier Generation, founded by the American Heart Association and the William J. Clinton Foundation to reduce prevalence of childhood obesity in the United States. The program is funded by the Robert Wood Johnson Foundation along with local, state, and philanthropic partners. This article addresses 2 questions: 1) how much improvement do schools make when HSP gives them hands-on training and technical assistance? and 2) which improvements are more prevalent? This study does not include the thousands of schools that have signed up exclusively for on-line participation in HSP. A companion article (11) addresses training and technical assistance (TTA) factors associated with school progress.

## Methods

### Study design

Schools recruited during the 2007-2008 and the 2008-2009 school years completed baseline and follow-up inventories of policy, practice, and environment indicators. Baseline was then compared with the most recent inventory completed by schools as of December 2010. HSP is a 4-year program, so this interim evaluation represents 2 snapshots of progress for schools at various stages in the program. Institutional review board approval was obtained from the RMC Corporation Human Subjects Protections Committee.

### The Healthy Schools Program

HSP builds schools' capacity to implement evidence-based policies and programs that promote and provide access to healthy foods and physical activity. HSP provides TTA at no cost, online tools for step-by-step practice standards and continuous improvement, and a national recognition program that recognizes schools reaching certain standards. HSP normally provides TTA for 4 years, although some schools complete TTA early. Regional HSP relationship managers recruit the schools, whose principals then designate representatives to undergo TTA. During a 4-year period, the relationship managers lead the school representatives through 9 highly structured train-the-trainer sessions that focus on a 6-step change process. These steps are 1) formation of a school wellness council; 2) completion of an assessment, the HSP Inventory; 3) local prioritization and action planning; 4) technical resource development and brokering; 5) implementation support; and 6) monitoring and evaluation of progress through updates to the HSP Inventory. Schools complete training at their own pace.

Through these sessions and other contacts, school representatives train relevant school personnel to achieve best practices in 8 content areas, described in the HSP Framework (www.healthiergeneration.org/schools.aspx?id=3470). These content areas are 1) school-level health policy, infrastructure, and systems; 2) school nutrition programs; 3) competitive foods and beverages (those sold outside of the school meals programs); 4) physical activity opportunities before, during, and after school; 5) physical education programs; 6) health education programs; 7) before- and after-school programs; and 8) school employee wellness programs. Training content depends on schools’ individual action plans, but relationship managers encourage schools to attempt new improvements over time.

Relationship managers contact schools between these sessions and arrange for TTA by 7 national content experts who tailor technical assistance to school needs and action plans. Through the HSP website, participating schools can access an online database containing more than 800 resources and 8 online topical tool kits aligned to the framework and vetted by the American Heart Association's Science Advisory Committee. School representatives receive a biweekly electronic newsletter with information about grant opportunities, new wellness resources, school successes, and current research.

### Program schools

We pilot-tested the HSP program in 224 schools in 13 states in 2006-2007 and performed factor analysis to refine the outcome measures. In the 2007-2008 and 2008-2009 school years, we recruited schools in these and 20 additional states and the District of Columbia. We recruited schools in the additional states because their adult obesity rate was 25% or higher, or they had the highest statewide obesity rates among children aged 10 to 17 years (12,13). The relationship managers targeted schools with predominantly low-income or racial/ethnic minority populations and recruited from feeder webs (elementary and middle schools that feed into 1 high school). Although we recruited individual schools, not school districts, 4 entire urban school districts also adopted HSP.

Recruitment consisted of signing a contract and attending an initial training session. Schools were HSP participants if, within 1 year of recruitment, they continued to take part in TTA, submitted an action plan, or submitted follow-up information about progress.

### Data collection

The HSP Inventory is an online tool that measures schools' progress over time (Appendix). Submission is voluntary and no incentives are provided, but we encourage school representatives to update their inventories at least annually to assess progress and realign priorities. The school representatives collected the necessary information from food service directors, physical education teachers, principals, and others. We chose online submission because it yielded data at a reasonable cost and provided schools with a tool for continuous quality improvement. School staff provide better evaluation information when feedback is useful to them for purposes such as quality improvement (14). Schools updated their inventories at their own pace. Most schools completed their baseline inventory when they started implementing HSP, and the median school had submitted its most recent follow-up inventory 6 months before December 2010 (range, 0-32 mo). The mean (standard deviation) number of months between baseline and follow-up inventories was 18.6 (8.6).

### Outcome measure

An expert panel comprising thought leaders in education, health, and health promotion in academia, government, and professional associations reviewed and approved inventory items. We revised some inventory items between 2007 and 2008 to reflect emerging research on best practices, but 83 items were common to both versions. The inventory collects information for 8 content areas; item responses in each content area are dichotomized and summed to create 8 content area index scores and a total index score. The 9 indices are policy and systems (6 items), school meals (17 items), competitive foods and beverages (12 items), physical activity outside of physical education (6 items), physical education (14 items), health education (10 items), before- and after-school programs (6 items), school employee wellness (12 items), and total (all 83 items). Reliability was excellent for the total index (Cronbach α = .90) and fair to good for 6 content areas (Cronbach α  = .63-.87). The indices for policy and systems and physical activity outside of physical education had lower Cronbach α (.57 and .52, respectively). In 2009, we directly assessed validity in 21 pilot-year schools that submitted a revised HSP Inventory. School meals and competitive foods and beverages could not be directly assessed. Agreement with the inventories ranged from 55% to 78%; discrepancies most often were due to out-of-date inventories.

### Data analysis

Using SPSS 18 (SPSS, Inc, Chicago, Illinois), we conducted *t* tests to compare the means of the indices for the baseline and follow-up inventories. To gauge the extent of change we supplemented the significance tests by examining Cohen's *d* effect sizes and percentage of schools improving in each content area. We used listwise deletion to handle missing data.

## Results

Relationship managers recruited 80% of the schools they approached to participate. Of 1,909 schools recruited, 1,514 (79%) continued or completed HSP (Table 1). Of participants, 42 completed the pilot-year's inventory at baseline and were ineligible for the study, leaving 1,295 (86%) of continuing and completed schools that provided both a baseline and follow-up inventory.

In both the baseline and follow-up inventory groups, the primary race/ethnicity of students at most schools was African American or Hispanic; most students were eligible for free or reduced-price lunches (Table 2). More than half the schools were in the South. We recruited urban schools in large numbers. A higher proportion of the schools that provided follow-up data were in the Midwest and South compared with the participating schools overall. Schools providing follow-up data also included more elementary schools, fewer schools in cities, and more rural schools than did overall HSP participants. All differences were significant.

The mean differences in the scores for baseline and follow-up inventories were significant for all content areas, and generated effect sizes of this magnitude are normally regarded as moderate to large (Table 3). The index for the total of all content areas showed the largest effect, followed by the school employee wellness content and school meals areas.

## Discussion

Schools that provided baseline and follow-up inventories to HSP made moderate to large changes in policies, practices, and environment. In contrast, recent national surveys of schools indicate few improvements to prevent obesity and little change in competitive foods and beverages or physical education (17,18). The program recruited 80% of the schools it approached, and these were schools with few resources but students at greatest risk of obesity. If these schools exhibit significant changes after receiving TTA, then it is likely that HSP will also benefit other schools.

We may be underestimating the extent of change since half the inventories were more than 6 months old. Sustainability is a question for further research. Nonresponse from 12% of eligible schools is of concern, but it is likely that they too made progress. In 2008, we audited progress in schools that participated exclusively online and did not complete inventories. The majority of those schools made improvements (data not shown).

The most common improvements were in the areas of school employee wellness and school meals. Employee wellness changes were easy to make, giving schools an early "win" to maintain momentum. The schools were also motivated to change school meals to respond to recent public demands. Less common were changes in physical education and policy and systems, which are most often controlled by the school districts. Schools also changed less in the area of physical activity outside of physical education. Lower reliability for the physical activity outside of physical education category and the policy and systems category may be partly responsible. The least common changes were in before- and after-school programs, which schools implement with community partners and which did not focus on the school day.

This program evaluation had several limitations. We could not validate information about school meals and competitive foods and beverages. Schools progressed through the program and provided data at their own pace, so the evaluation is a snapshot of accumulated changes. In the absence of a control group we cannot assert any causal relationship between HSP and school progress. These schools might have made progress in the absence of HSP, but that is unlikely given national surveys that indicate little progress (17,18).

Schools participating in Healthy Schools Program TTA improved policies, practices, and environments related to childhood obesity prevention. These findings help to identify the TTA needed to implement the 2010 Healthy, Hunger-Free Kids Act (4), which requires minimum standards to promote less calorie-dense offerings in all foods sold in school and requires changes in schools to promote both physical activity and healthy eating.

## Acknowledgments

This evaluation was supported by grant 61099 from the Robert Wood Johnson Foundation.

## Figures and Tables

**Table 1.** School Participation Status in the Healthy Schools Program, 2007-2010

Status in 2010	Initial Year^a^		Total

	2007-2008	2008-2009	
Continuing^b^	549	932	1,481
Inactive^c^	38	37	75
Dropped^d^	85	138	223
School closed	36	30	66
Completed program^e^	23	10	33
Transitioned to online^f^	0	31	31
Total recruited	731	1,178	1,909

a Initial year is based on the date of initial sessions and memorandum of understanding.

b Schools that continued training and technical assistance and provision of inventories or action plans through December 2010.

c Schools that did not continue training and technical assistance or update an inventory or action plan in a year.

d Schools that chose not to continue the Healthy Schools Program.

e Schools that completed the Healthy Schools Program.

f Schools in Alaska that lost their relationship manager and transitioned to online participation exclusively.

**Table 2.** Characteristics of Schools Participating in the Healthy Schools Program (HSP) and Schools Providing Both Baseline and Follow-up HSP Inventory Data, 2007-2010^a^

Characteristic	Number and Percentage With Characteristic					

	2007-2008		2008-2009		Total	

	Recruited Schools (n = 731), n (%)	Data Provided (n = 501), n (%)	Recruited Schools (n = 1,178), n (%)	Data Provided (n = 794), n (%)	Recruited Schools (n = 1,909), n (%)	Data Provided (n = 1,295), n (%)
**School level**						
Elementary	438 (60)	306 (61)	735 (62)	518 (65)	1,173 (61)	824 (64)
Middle	138 (19)	99 (20)	186 (16)	121 (15)	324 (17)	220 (17)
High	113 (15)	77 (15)	152 (13)	101 (13)	265 (14)	178 (14)
Other/missing	42 (6)	19 (4)	105 (9)	54 (7)	147 (8)	73 (6)
**Students eligible for free or reduced-price lunch, %**						
0-24	51 (7)	16 (3)	74 (6)	47 (6)	125 (7)	63 (5)
25-49	135 (18)	98 (20)	173 (15)	118 (15)	308 (16)	216 (17)
50-74	171 (23)	134 (27)	356 (30)	245 (31)	527 (28)	379 (29)
75-100	336 (46)	234 (47)	428 (36)	285 (36)	764 (40)	519 (40)
Missing	38 (5)	19 (4)	147 (13)	99 (13)	185 (10)	118 (9)
**Primary race/ethnicity**						
White	211 (29)	133 (27)	432 (37)	312 (39)	643 (34)	445 (34)
African American	268 (37)	206 (41)	372 (32)	245 (31)	640 (34)	451 (35)
Hispanic	200 (27)	135 (27)	242 (21)	171 (22)	442 (23)	306 (24)
Other	20 (3)	10 (2)	52 (4)	27 (3)	72 (4)	37 (3)
Missing	32 (4)	17 (3)	80 (7)	39 (5)	112 (6)	56 (4)
**School locale**						
City	284 (39)	206 (41)	477 (41)	281 (35)	761 (40)	487 (38)
Suburb	201 (28)	111 (22)	298 (25)	230 (29)	499 (26)	341 (26)
Small town	83 (11)	69 (14)	125 (11)	97 (12)	208 (11)	166 (13)
Rural	139 (19)	102 (20)	223 (19)	157 (20)	362 (19)	259 (20)
Missing	24 (3)	13 (3)	55 (5)	29 (4)	79 (4)	42 (3)
**Region^b^**						
Northeast	152 (21)	78 (16)	191 (16)	122 (15)	343 (18)	200 (15)
Midwest	200 (27)	155 (31)	235 (20)	162 (20)	435 (23)	317 (25)
South	338 (46)	260 (52)	636 (54)	453 (57)	974 (51)	713 (55)
West	41 (6)	8 (2)	116 (10)	57 (7)	157 (8)	65 (5)

a Source: National Center for Educational Statistics (15).

b Region is based on US Census categorization (16).

**Table 3.** Comparison of Baseline and Follow-Up Scores for Inventory Content Areas in Schools That Completed a Baseline and Follow-Up Inventory (n = 1,295), Healthy Schools Program, 2007-2010

**Content Area**	No. of Items	Baseline, Mean (SD)^a^	Follow-up, Mean (SD)^a^	Cohen's *d* Effect Size	Percentage That Improved^b^
Total	83	40.4 (10.6)	47.9 (11.5)	0.83	79
School employee wellness	12	4.1 (1.9)	5.5 (2.2)	0.63	58
School meals	17	11.1 (3.1)	12.6 (2.9)	0.60	56
Health education	10	4.3 (2.5)	5.3 (2.4)	0.50	49
Competitive foods and beverages	12	3.9 (2.8)	5.1 (2.9)	0.48	48
Physical education	14	7.2 (2.3)	7.9 (2.0)	0.42	44
Physical activity outside of physical education	6	2.7 (1.5)	3.2 (1.5)	0.49	41
Policy and systems	6	4.2 (1.4)	4.7 (1.2)	0.51	39
Before and after school programs	6	2.9 (1.9)	3.6 (1.9)	0.41	37

Abbreviation: SD, standard deviation.

a Baseline and follow-up scores are sums of the desired responses.

b Percentage of schools that improved on 1 or more items. All gains were significant (*P* < .001).

## Appendix. Healthy Schools Inventory

### Items Common to 2007-2008 and 2008-2009 Inventories

This appendix is available for download as a Microsoft Word file [DOC - 203k]
