# Supplementary material for: Evaluation of the Healthy Schools Program: Part I. Interim Progress
Source: Prev Chronic Dis. 2012 Mar 1;9:E65. doi: 10.5888/pcd9.110106 (PMC3366696; doi:10.5888/pcd9.110106)
Supplement: Supplementary file 1 [file 11_0106_01.doc]

Healthy Schools Inventory

Items Common to 2007-2008 and 2008-2009 Inventories

**Policy/Systems**

1. Our district has adopted a wellness policy containing the elements required by the 2004 Congressional Child Nutrition reauthorization.

- Yes
- No

1. All parents and guardians have the opportunity to provide meaningful input into the development and implementation of school health and wellness activities.

- Yes
- No

1. The following policy/systems features exist in our school (please mark all that apply):

- Drinking water is available to all students free of charge at all times during the school day
- School wellness is a standing agenda item on the site council and/or parent group meetings
- Students have the opportunity to provide meaningful input into the development and implementation of school health and wellness activities
- School grounds are open to students, their families and the community for access to physical activity

**School Meals Programs**

1. School participates in the National School Breakfast and Lunch Programs or in independent breakfast and lunch programs that meet USDA nutrition standards

- Yes
- No

1. The National School Breakfast and Lunch Programs or the independent meals programs meet USDA access standards with a plan in place to avoid “overt identification” of students who qualify for free or reduced-price meals

- Yes
- No

1. School breakfast and lunch programs meet USDA School Meals Initiative (SMI) standards for reimbursable meals

- Yes
- No

1. Annual training, covering techniques such as reducing fat and sodium in food preparation, and portion control, is completed by 100% of food service staff who prepare and serve meals

- Yes
- No

1. The following are done as a part of our school meals program (please mark all that apply):

- Breakfast and lunch menus are in alignment with applications for free and reduced-price meals by way of being printed/available in the language(s) that parents primarily speak
- The school conducts yearly taste tests of foods that are representative of the variety of religions and cultures that make up the school student community
- The cafeteria uses an electronic point-of-sale system that protects low-income students participating in the free or reduced-price meal program from being stigmatized

1. Our school offers only whole grains daily at breakfast and lunch.

- Yes
- No

1. The following school meals program features exist in our school (please mark all that apply):

- Offers only 1%, ½% or fat-free milk (flavored or unflavored; flavored milk must contain no more than 150 calories per 8 oz.)
- Half of all grains offered daily, at breakfast and lunch, are whole grains
- At least one fruit (fresh, canned or frozen in fruit juice or light syrup) is offered at breakfast
- Offers at least four non-fried, no-added-sugar fruit and/or vegetable options daily (salad can serve as one of the four)
- Offers at least one low-fat entree choice at lunch with ≤ 35% calories from fat, ≤ 10% calories from saturated fat, 0 g trans fat and ≤ 480 mg sodium
- Uses only unsaturated (no more than 1 g saturated fat), zero trans fat oils during on-site (post-manufactured) food preparation
- Serves only non-fried food products (food products that have not been pre-fried, flash fried, or par-fried during the manufacturing process) and uses no deep fat frying in food preparation
- Offers non-fried fish at least one time per week
- Offers only lean protein products such as lean red meat, skinless poultry, lean deli meats, fat-free or low-fat cheese, beans, tofu, etc. (Lean: less than 10 g fat, 4.5 g or less saturated fat, and less than 95 mg cholesterol per serving and per 100 g.)

**Competitive Foods& Beverages**

1. All beverages *offered* for sale to students outside of the school meals program during the regular and extended school day meet or exceed the Alliance School Beverage Guidelines.

- Yes
- No
- No beverages are sold in our school

1. Our school has created an inventory of all competitive foods currently offered and taken the following actions (please mark all that apply):

- Completed an inventory of all competitive foods currently offered in vending machines, on a la carte lines, as fundraisers, and school stores and on snack carts to identify which meet the Alliance Competitive Foods Guidelines
- Created a list of competitive foods available from vendor(s) that meet the Alliance Competitive Foods Guidelines
- Developed a written policy stating that all competitive foods will be compliant with the Alliance Competitive Foods Guidelines within 12 months and sent this policy to parents and guardians
- Ensured all new Requests for Proposals and/or Requests for Quotes that contain competitive foods and are issued during this school year (even if effective for future school years) include only competitive foods that meet the Alliance Competitive Foods Guidelines
- Lowered the price of compliant competitive foods and raised the price of non-compliant foods in all areas where competitive foods are sold
- Substituted at least two non-compliant food fundraisers with non-food alternatives or with only products that meet the Guidelines
- Conducted one or more initiatives with an evaluation component to engage students in leading change toward healthier competitive foods at the school
- Conducted a marketing campaign with evidence of input from students, school staff, administration and food service staff to promote nutritious snack choices in all areas where competitive foods are sold

1. All competitive foods *offered* for sale to students outside of the school meal program during the regular and extended school day meet or exceed the Alliance Competitive Foods Guidelines.

- Yes
- No
- No food is sold outside the reimbursable meals program

1. With the exception of a maximum of two times per year, all beverages *served* to students outside of the school meals program during the regular and extended school day, including school and classroom parties, meet the Alliance School Beverage Guidelines.

- Yes
- No

1. With the exception of a maximum of two times per year, all competitive foods *served* to students outside of the school meals program during the regular and extended school day, including school and classroom parties, meet the Alliance Competitive Foods Guidelines

- Yes
- No

**Health Education**

1. Our school requires that every student enrolled in the *Kindergarten through 5^th^ grades* receives skills-based instruction on healthy eating and physical activity as part of a dedicated comprehensive health education program.

- Our school does not contain any of these grades
- Yes
- No

1. Our school requires that every student enrolled in the *6^th^ through 8^th^ grades* receives skills-based instruction on healthy eating and physical activity as part of a dedicated, stand alone, term-long health education course, or the equivalent.

- Our school does not contain any of these grades
- For at least one term during one year
- For at least one term during two years
- For at least one term during each year
- Our school does not require or requires less than one term of Health Education

1. Our school requires that every student enrolled in the *9^th^ through 12^th^ grades* receives skills-based instruction on healthy eating and physical activity as a part of a dedicated, stand alone, term-long health education course.

- Our school does not contain any of these grades
- For at least one term
- For the equivalent of two terms
- For the equivalent of three terms
- Our school does not require or requires less than one term of Health Education

1. The following are true of the Health Education program at our school (please mark all that apply):

- Planned healthy eating and physical activity instruction is aligned to the national/state health education standards
- Health education is taught by trained teachers at the elementary school level or teachers certified/licensed in health education at the secondary level
- All teachers who teach health education receive annual professional development on effective practices for health education, including physical activity and healthy eating, for a minimum of three contact hours at the elementary level and eight contact hours at the middle and high school levels
- Healthy eating and physical activity messages are integrated into other subject areas
- At the middle and high school levels, health education electives offering additional instruction on healthy eating and physical activity are offered
- Health education curriculum instructional strategies and examples are inclusive of the diversity of the student population
- All students are assessed in health education and results are reported on the report card every term that health education is offered

**Physical Activity**

1. Our school provides the following physical activity opportunities for students (please mark all that apply):

- All students have the opportunity to participate in physical activity breaks on a daily basis
- Our school has an annual plan for integrating physical activity into most subject areas
- At the elementary school level, at least 20 minutes of recess is offered daily
- Our school offers a range of competitive physical activity opportunities (including intramural or interscholastic sports) before or after the school day
- Our school offers a range of non-competitive physical activity opportunities aimed at engaging students in fun, recreational and life-long learning opportunities before or after the school day
- Our school has a plan in place to promote safe walking and bicycling to/from school

**School Employee Wellness**

1. Our school has (please mark all that apply):

- Identified a school employee wellness leader or committee
- Obtained administrator’s support for development of a school employee wellness program
- Conducted a school employee wellness needs assessment with staff
- Developed a written school employee wellness action plan based on the results of the needs assessment that at a minimum includes opportunities related to physical activity and healthy eating

1. Our school is implementing our school employee wellness action plan that at a minimum includes opportunities related to physical activity and healthy eating.

- Our school does not have a school employee wellness action plan
- Yes
- No

1. Our school’s school employee wellness action plan is evaluated annually.

- Our school does not have a school employee wellness action plan
- Yes
- No

1. Our school’s school employee wellness action plan includes opportunities related to (please mark all that apply):

- Our school does not have a school employee wellness action plan
- Weight Management
- Health Screenings
- Stress Management
- Tobacco Cessation

1. Food and beverages sold and served in the staff lounge and at school-sponsored staff functions meet at least the Alliance High School Beverage and Competitive Food Guidelines.

- Yes
- No

**Physical Education**

1. Please mark the number of minutes of physical education that your school requires for all students enrolled in *Kindergarten through 5^th^ grades*:

- Our school does not contain any of these grades
- Less than 60 minutes per week
- 60 - 89 minutes per week
- 90 -149 minutes per week
- 150 minutes or more per week

1. Please mark the number of minutes of physical education that your school requires for all students enrolled in the *6^th^ through 8^th^ grades*:

- Our school does not contain any of these grades
- Less than 90 minutes per week
- 90 - 134 minutes per week
- 135 - 224 minutes per week
- 225 minutes or more per week

1. Please mark the number of years your school requires for all students enrolled in the 6th through 8th grades to participate in physical education:

- Our school does not contain any of these grades
- None
- One year
- Two years
- All years

1. Please mark the number of years of physical education that your school requires for all students enrolled in the *9^th^ through 12^th^ grades*:

- Our school does not contain any of these grades
- Less than ½ year
- At least the equivalent of ½ year, but less than 1 year
- At least the equivalent of 1 year, but less than 1.5 years
- At least the equivalent of 1.5 years

1. If the school is a middle or high school, the school offers opportunities for all students in all grades to enroll in physical education courses beyond what is required.

- Our school is an elementary school
- Yes
- No
- Our school requires PE for all students at all grades so additional PE is not offered

1. If the school is an elementary school, physical education is taught by:

- Our school is a middle or high school
- Appropriately trained classroom teachers
- Licensed or certified physical educators
- Our school does not teach physical education

1. If the school is a middle or high school, physical education is taught by:

- Our school is an elementary school
- Appropriately trained classroom teachers
- Licensed or certified physical educators
- Our school does not teach physical education

1. The following are true of the Physical Education programs at our school (please mark all that apply):

- Physical education is based on a sequential curriculum map that is aligned to the national and state (if applicable) standards for physical education
- Students are moderately to vigorously active for at least 50% of physical education class time
- All students are assessed in mastery of skills and content in physical education and results are on the report card every term that physical education is required
- The student/teacher ratio in physical education is comparable with other classes at all grade levels
- Physical education classes are appropriately modified or adapted to promote the participation of all students in physical education (in particular students with chronic health conditions and special needs)
- All teachers who teach physical education receive annual professional development on effective practices in physical education for a minimum of eight contact hours
- Physical education instructional strategies and other practices support needs of the diversity of student population

**Before & Afterschool Programs**

1. The following is true of before and afterschool programs at our school (please check all that apply):

- Our school does not offer before or afterschool programs
- Before and afterschool program offerings dedicate at least 20 percent of their time to physical activity
- At the elementary and middle school levels, a healthy snack is offered as part of the After School Snack Program reimbursed through the USDA or an independent meal program that meets the Alliance Competitive Food and Beverage Guidelines
- Before and afterschool programs offer a variety of physical activity opportunities that reflect the diversity and needs among students, families and the community
- At the elementary and middle school levels, snacks offered are healthy food and beverage selections that reflect the diverse demographics of the school community
- Our school encourages students to connect with physical activity opportunities in the community
- All before and afterschool program staff participate in annual professional development on the importance of and strategies for including physical activity and healthy eating as elements of their programs
